# Supplementary material for: CCR9/CXCR5 Co-Expressing CD4 T Cells Are Increased in Primary Sjögren’s Syndrome and Are Enriched in PD-1/ICOS-Expressing Effector T Cells
Source: Int J Mol Sci. 2023 Jul 26;24(15):11952. doi: 10.3390/ijms241511952 (PMC10418442; doi:10.3390/ijms241511952)
Supplement: Supplementary file 1 [file ijms-24-11952-s001.zip › ijms-2390078-supplementary.pdf]

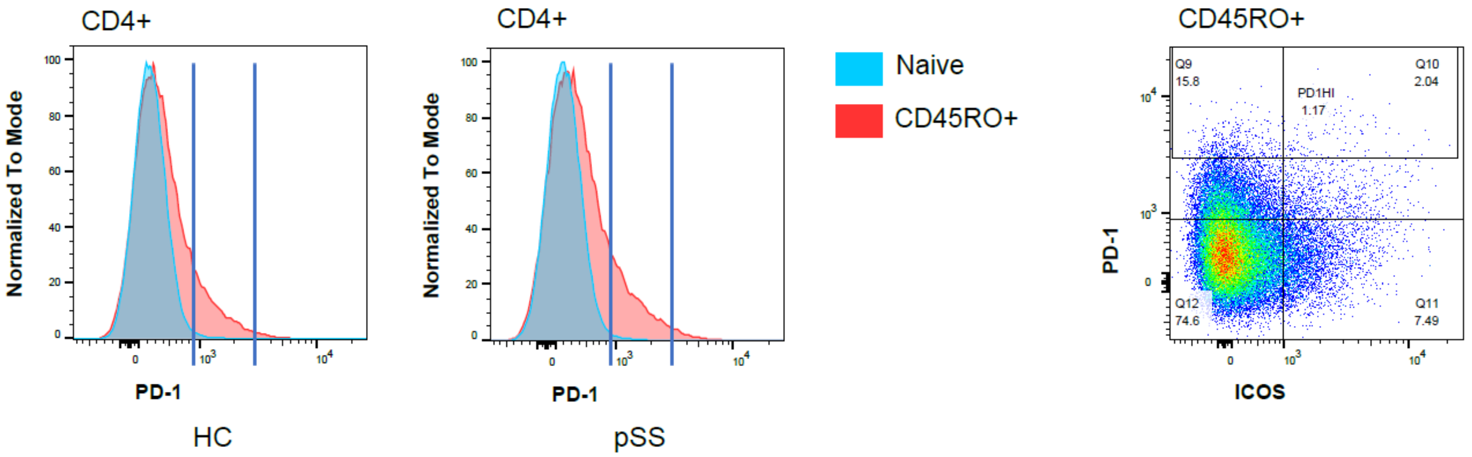

Supplementary Figure S1. PD-hi staining

Representative histograms of PD-1 staining in naive and memory (CD45RO+) CD4 T cells from one HC and one pSS patient. Vertical lines in histograms indicate gating of PD-1 negative, PD-1^{intermediate}, and PD-1^{hi} populations, as can also be seen in representative ICOS/PD-1 dotplot from memory CD4 T cells. HC: healthy control; pSS: primary Sjögren's syndrome; ICOS: inducible T cell co-stimulator; PD-1: programmed death-1.

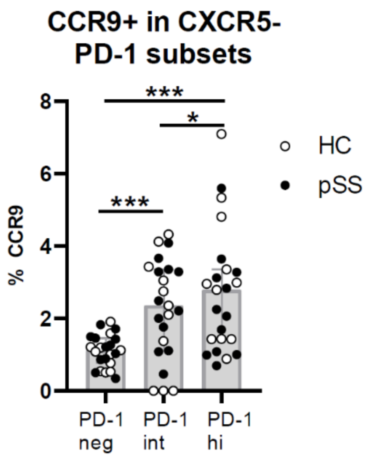

Supplementary Figure S2. The percentage of CCR9-expressing cells is associated with PD-1 expression

CCR9 expression is evaluated on total (incl naïve and memory) CXCR5- CD4 T cells with negative, intermediate, and high PD-1 expression. HC: healthy control; pSS: primary Sjögren's syndrome. CCR9: C-C motif chemokine receptor 9; PD-1: programmed death-1; neg: negative; int: intermediate; hi: high. *, *** indicates p-value <0.05, 0.001, respectively.

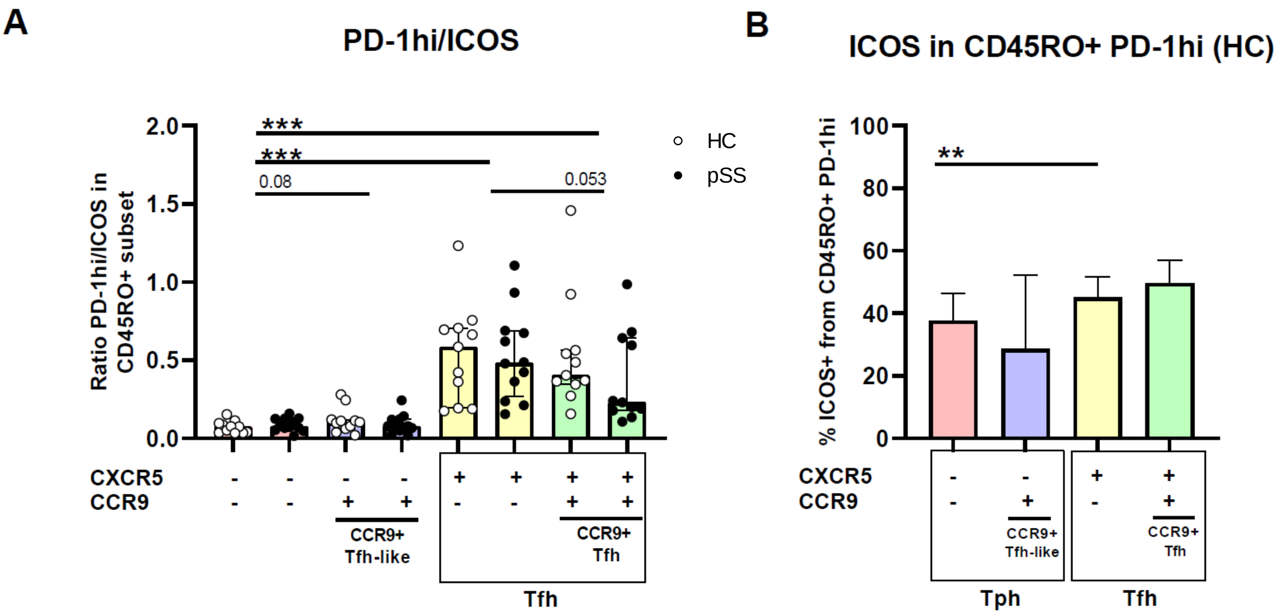

Supplementary Figure S3. CXCR5-expressing cells have a higher PD-1^{hi}/ICOS ratio. Increased ICOS expression in HC was only seen for CXCR5-expressing Tfh cells compared to CXCR5-CCR9- cells.

(**A**) Ratios of PD-1hi/ICOS in memory population of all four CXCR5/CCR9-defined cell subsets in pSS patients and HC. (**B**) Comparison of ICOS expression between the memory PD-1^{hi} CXCR5-CCR9- subset compared to the three other subsets in HC. Plots show medians plus interquartile ranges. HC: healthy control; pSS: primary Sjögren's syndrome. Tph: T peripheral helper cell (memory CXCR5-PD-1^{hi} CD4 T cell); CCR9+ Tfh-like: C-C motif chemokine receptor 9-expressing T follicular helper-like cell (CXCR5-CCR9+ CD4 T cell); ICOS: inducible T cell co-stimulator; PD-1: programmed death-1; **, *** indicates p-value <0.01, 0.001, respectively.

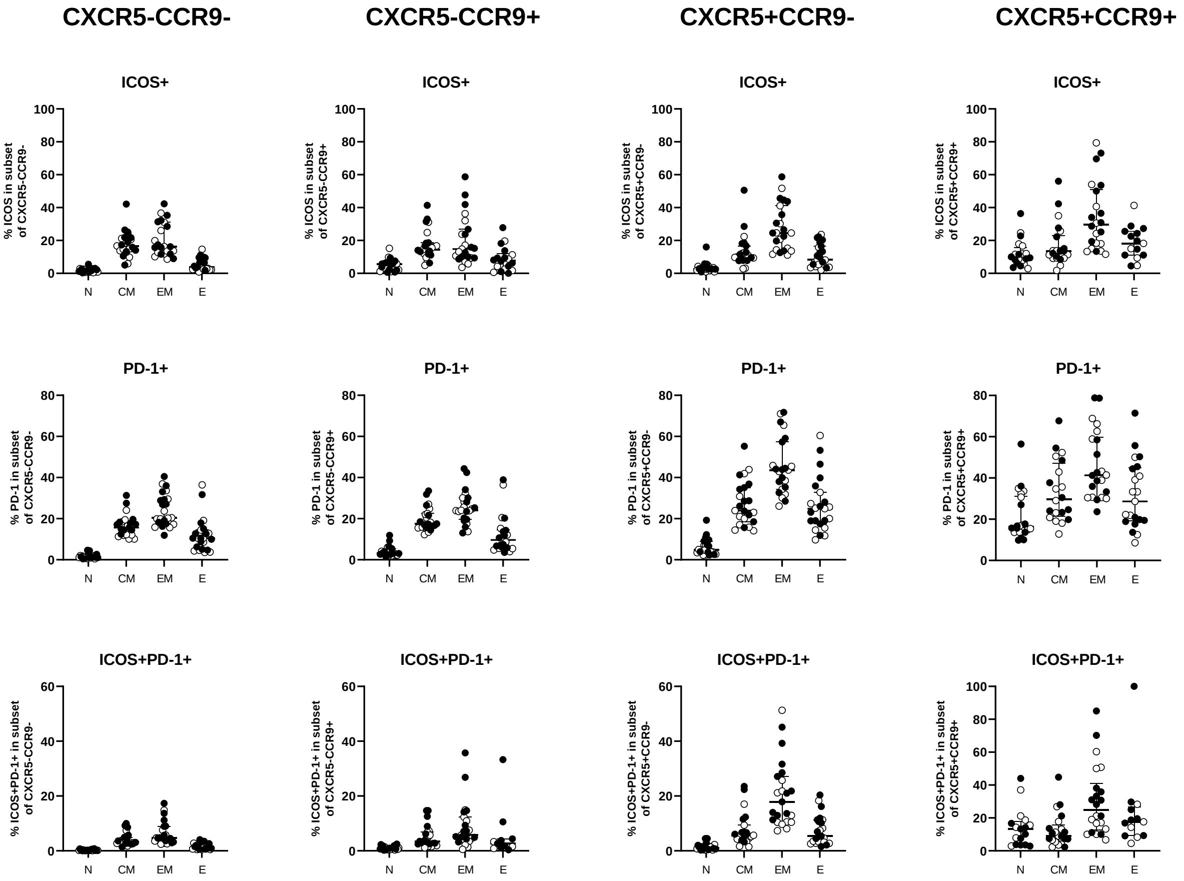

Supplementary Figure S4. ICOS+, PD-1+, and ICOS+PD-1+ are significantly lower in naive cells in all CXCR5/CCR9-defined cell subsets.

Expression of ICOS, PD-1, and ICOS+PD-1+ in naive (N), central memory (CM), effector memory (EM), and effector (E) cells in all four CXCR5/CCR9-defined cell subsets. All comparisons between N cells and CM/EM/E cells are significant within the same CXCR5/CCR9-defined cell subset ($p<0.01$), except for ICOS expression between N and E in CXCR5-CCR9+ and CXCR5+CCR9+ cells ($p=0.19$ and $p=0.43$, respectively), and ICOS+PD-1+ expression between N and E cells in CXCR5+CCR9+ subset ($p=0.21$). Plots show medians plus interquartile ranges. Naive (N): CD27+CD45RO-; Central memory (CM): CD27+CD45RO+; Effector memory (EM): CD27-CD45RO+; Effector (E): CD27-CD45RO-.

Supplementary Table S1. Antibodies used for flow cytometry.

Marker	Fluorochrome	Company	Clone
**CD3**	AF700	Sony Biotechnology	UCHT1
**CD4**	BV785	Biolegend	RPA-T4
**CD8**	PE	Biolegend	RPA-T8
**CCR9**	APC	Biolegend	L053E8
**CXCR5**	PerCP-Cy5.5	Biolegend	J252D4
**CD27**	BV510	BD Biosciences	L128
**CD45RO**	PE-Cy7	BD Biosciences	UCHL1
**ICOS**	FITC	Biolegend	C398.4A
**PD-1**	BV711	BD Biosciences	EH12.1
**Fixable Viability Dye**	eFluor™ 780	eBioscience	n.a.
